# Development of a serious game-based cognitive rehabilitation system for patients with brain injury

**DOI:** 10.1186/s12888-023-05396-2

**Published:** 2023-11-29

**Authors:** Meysam Rahmani-Katigari, Fatemeh Mohammadian, Leila Shahmoradi

**Affiliations:** 1 Department of Health Information Management, Saveh University of Medical Sciences, Saveh, Iran; 2 Department of Psychiatry, Roozbeh Hospital, Tehran University of Medical Sciences, Tehran, Iran; 3 Department of Health Information Management, School of Allied Medical Sciences, Tehran University of Medical Sciences, Tehran, Iran

**Keywords:** Serious game, Cognitive rehabilitation, Attention impairment, Video game, Brain injury

## Abstract

**Background:**

Traumatic brain injury (TBI) resulting from a forceful impact to the head can cause severe functional disabilities, with cognitive impairment being a major hindrance to patients' return to daily life. Encouraging patients to engage in rehabilitation programs consistently poses a significant challenge for therapists. To address this issue, gamification has gained momentum as an effective approach. This study aims to develop a serious game-based cognitive rehabilitation system tailored for patients with brain injury.

**Methods:**

The study included four stages. Initially, the requirements were analyzed through focus groups. Then the system structure and game content were discussed and was agreed as a conceptual model. In second stage, the system design was drawn using various modeling diagrams. In third stage, a system prototype was developed using the Unity game engine and C# programming. Finally, a heuristic evaluation method was employed to assess usability.

**Results:**

Based on the focus group meetings with seven participants, a conceptual model of the system structure and game content was designed. Game's interface was developed for both the therapist and patient versions. The focus groups determined a 2D casual gaming genre with a postman character and 10 missions on the smartphone platform. For example, in the first mission, the postman must move from mailboxes 1 to 10 and pick up the letters. This is according to Trail Making Test task. The 16 tasks in different subcategories of attention were selected to make these missions. The usability evaluation highlighted privacy, help and documentation, and aesthetic and minimalist design as the areas with the highest percentage of problems.

**Conclusions:**

Cognitive rehabilitation is vital in facilitating patients' faster return to daily routines and enhancing their quality-of-life following brain injury. Incorporating a game-based system provides patients with increased motivation to engage in various cognitive exercises. Additionally, continuous monitoring by specialists ensures effective patient management. The game-based system offers different game stages to strengthen and rehabilitate attention in patients with brain injury. In the next step, the clinical effects of this system will be evaluated.

## Introduction

Traumatic brain injury (TBI) is a result of blunt force trauma or penetrating head trauma that disrupts brain function, leading to significant physical, cognitive, psychological, and social disabilities [1]. These injuries commonly occur due to traffic accidents, sports events, falls, conflicts, or fights [2]. The severity of brain damage can range from impaired consciousness to severe disability or even death [2]. TBI affects approximately 50 to 60 million people worldwide annually, with a higher prevalence in developing countries [3]. While physical impairments are often associated with brain injuries, it is now well-established that cognitive, emotional, and behavioral functioning pose the most challenging obstacles to individuals reintegrating into interpersonal interactions, school, and the workplace [4].

Among the various consequences of TBI, attention deficits are particularly prevalent, affecting about 80% of patients [4]. Attention refers to the allocation of neural processing resources. One of the models that is widely used in the division of types of attention is Sohlberg and Mateer's hierarchical clinical model [5]. Five different types of attention are described in this model. 1) Focused attention: is the ability to separately respond to visual and auditory stimuli. 2) Sustained attention: refers to the ability to maintain a constant behavioral response during repeated and continuous activity. 3) Selective attention: It refers to the capacity to maintain a behavioral or cognitive set in the face of confusing or competing stimuli. 4) Alternating attention: It is the capacity of mental flexibility that allows people to shift their attention center and move between tasks that have different cognitive requirements. 5) Divided attention: It is the highest level of attention and refers to the ability to simultaneously respond to multiple tasks [6]. Due to the significant impact of attention deficits on other cognitive functions, researchers have focused on developing effective treatments for this impairment [7]. It has been demonstrated that the brain can undergo repair through repetitive, intensive, and task-oriented training following damage [8]. Cognitive rehabilitation, behavior modification, psychological management, education, and individual and family counseling are among the primary methods of treatment in the rehabilitation of TBI patients [9]. Many researchers have emphasized the importance of cognitive rehabilitation in reducing behavioral and cognitive consequences and improving independence and quality of life [10–12]. The two main categories of cognitive rehabilitation include the traditional and the computer-based method [13]. Traditional methods refer to tasks that are used without the use of computers to perform. Anyone with a little creativity can design and perform such tasks at home. These are presented on paper [14]. Despite the positive effects of traditional or non-computerized rehabilitation methods, they have problems such as being boring, not having enough motivation to continue [14].

One of the key challenges faced by therapists is how to motivate patients to consistently engage in rehabilitation programs [15, 16]. Additionally, it is crucial to consider the individual differences in the deficits of TBI patients for evaluation, development, and rehabilitation planning [17]. In recent decades, computer-based cognitive rehabilitation programs have gained recognition for their positive effects on various cognitive deficits [18–20]. Such interventions offer advantages such as cost reduction, personalized treatment, availability, immediate feedback, quantitative outcomes, and significant therapeutic benefits [21].

Among computer-based interventions, the use of serious game-based tools in rehabilitation is rapidly growing [22]. Serious games have long been used in physical rehabilitation, but their application in cognitive rehabilitation is less common [23]. Serious gaming is a relatively new term that refers to those computer games that have some other primary purpose than entertainment [24]. Traditional rehabilitation methods often involve repetitive and monotonous activities, discouraging patients from completing the tasks. However, with the widespread familiarity of digital environments, particularly mobile phones, health professionals are increasingly interested in utilizing mobile-based video games for rehabilitation purposes [25, 26].

Video games offer several advantages, including increased rehabilitation quality and efficiency, overcoming the monotony associated with traditional rehabilitation, providing different levels of therapeutic interventions, easy distribution through the internet, personalization options, and the ability to be used at home and in remote areas [27, 28]. Baranyi et al., have developed serious game as MyDailyRoutine for patients suffering from cerebral dysfunction. Making a cup of coffee was an example of the games in this study [29]. RehabCity is a city simulator game that includes street, moving cars, parks, sideways, buildings, and etc. Users have to perform the daily tasks of life in this city [30]. Gamito et al., developed a serious virtual reality-based game with cognitive training for memory and attention that included daily activities [31]. While computer games for cognitive rehabilitation, specifically in the area of attention, have gained popularity outside of Iran, the literature review indicates limited usage of such programs within Iran. This can be attributed to factors such as the lack of suitable games, reliance on foreign games in English, and limited familiarity among therapists and patients. Therefore, the purpose of this study is to design and create a serious game-based attention rehabilitation system specifically tailored for TBI patients in Iran.

## Methods

**Fig. 1:**
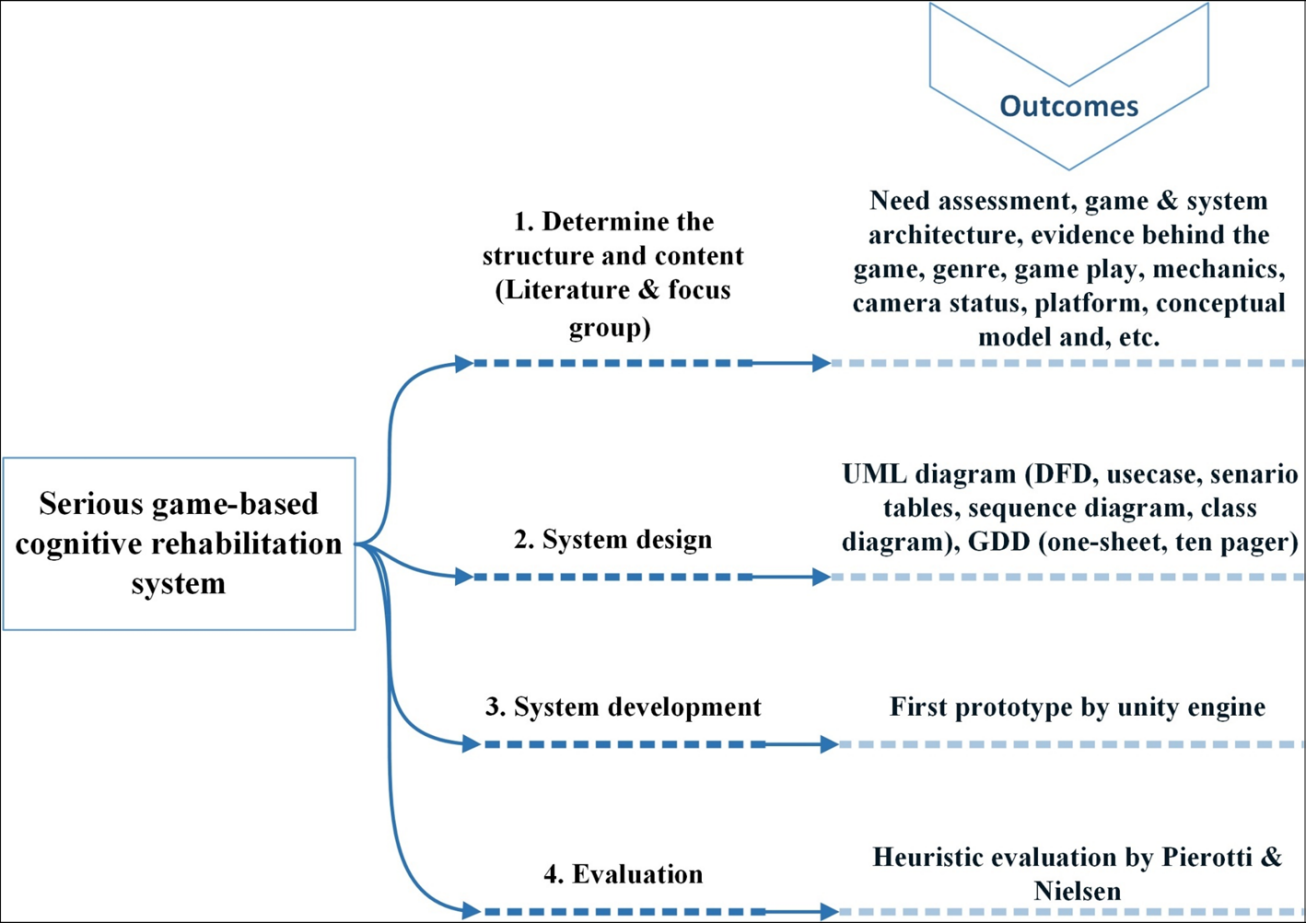
The stages of system development, along with the outcome of each stage. Note: GDD: Game Development Documents; DFD: Data Flow Diagram; UML: Unified Modeling Language

This research employed a developmental study design, utilizing both quantitative and qualitative methods, conducted from 2020 to 2022. The study consisted of four main stages, as illustrated in Fig. 1.

The first stage of the study involved conducting a comprehensive literature review to identify available games for attention rehabilitation and examine their features. After writing the protocol and its approval by the authors, six electronic databases PubMed, Web of Science, Embase, Scopus, IEEE, Cochrane, in addition to manual searches were used to conduct the search. The findings from this literature review have already been published [32]. Then, the focus group method [33] was employed to analyze users' requirements and determine the system structure and content of the games and system. The focus group consisted of two experts in cognitive rehabilitation with a minimum of 5 years of experience, two experts in health informatics, and three experts in game development. A facilitator was present to manage the meetings. Two 90-min sessions were conducted, allowing for face-to-face interaction, while also providing a virtual environment for participants who were unable to attend in person. All the participants were present in both meetings either virtually or in person.

The focus group meetings were conducted in the conference hall of Roozbeh Hospital, which is affiliated with Tehran University of Medical Sciences. The conference hall was equipped with a computer system, internet access, and an audio and video recording system to facilitate the discussions. At the beginning of each meeting, the facilitator provided an introduction to the topic and purpose of the meeting to ensure that all participants were familiar with the content. The predetermined questions and issues were then raised and discussed. Participants were free to comment, discuss and make suggestions on the topic. The questions used in this stage were unstructured and aimed to gather insights on various dimensions related to attention deficits and rehabilitation. These questions included:

1) In what dimensions do patients with attention deficits need to improve? 2) What trainings and methods are appropriate for attention rehabilitation in these patients? 3) What kind of games are suitable for these patients? 4) What capabilities should the system have? 5) What should the content of the games include? 6) What should be chosen as the main character in the game?

To analyze the data obtained from the focus group meetings, the researchers employed framework analysis, which involved five main steps: familiarization, identification of thematic framework, indexing, charting, mapping, and interpretation [34]. After the meetings, the recorded interviews were transcribed and implemented in Microsoft Word software version 2016. The transcripts were read multiple times to ensure a thorough understanding of the content. Any ambiguities or unclear points were addressed and resolved based on the researcher's notes taken during the interviews. Once the researcher became familiar with the scope and diversity of the material, key concepts were identified, and a thematic framework was established. This framework served as the basis for the analysis and coding of the interviews. During the indexing stage, coding was applied to categorize and organize the data. The results of the coding process were then used to create a conceptual model that captured the main themes and findings derived from the focus group discussions [34].

In the system design stage, various tools and software were utilized to create a comprehensive understanding of the system and facilitate game development. To better understand the system, a data flow diagram (DFD) [35] and unified modeling language (UML) diagrams, including use case, sequence, and scenario tables, were drawn using Microsoft Visio 2016 software [36]. These diagrams helped visualize the flow of data and interactions within the system. In addition, Game Development Documents (GDD) were documented using Microsoft Word 2016 software. The GDD served as the main reference for the game development process, outlining the game's features, mechanics, and overall design [37]. During the system development stage, characters and graphic components of the user interface were designed and created using Adobe Photoshop version 2018. These visual elements added to the overall aesthetics and user experience of the game.

In the third stage, system prototype was developed using the Unity game engine and C# programming language [38]. PHP language was utilized to establish a connection between the system and the server. To manage the MySQL database, PHPMyAdmin version 5.2 was used.

In the final stage of the research, the usability of the developed system was evaluated using the heuristic method. The evaluation process involved the use of the 13-item version of Pierotti & Nielsen's checklist [39], which assesses various usability principles. The evaluation was conducted by five experts who possessed the necessary skills to evaluate health systems and had undergone a familiarization stage. These experts systematically assessed the system's usability based on the checklist items. The data obtained from the checklists were analyzed using descriptive statistics, with Microsoft Excel version 2016 being used for this purpose. The results were then presented in the form of tables and graphs, providing a clear overview of the system's strengths and weaknesses. The identified defects and problems mentioned in the checklists will be thoroughly reviewed and addressed to improve the system for the next version. This iterative process ensures that the system evolves and becomes more user-friendly and effective.

The checklist used in the evaluation covers various usability principles, including visibility of system status, a match between the system and the real world, user control and freedom, consistency and standards, error prevention, recognition rather than recall, flexibility and minimalist design, aesthetic and minimalist design, help and documentation, skills, pleasurable and respectful interaction with the user, and privacy. It is worth noting that the questionnaire used in this research was translated by Rezaei Hachesu et al., and its validity and reliability have been confirmed [40]. This ensures that the evaluation process is based on a reliable and validated instrument. The use of the 13-item version was due to the existence of the localization version and its availability. The questionnaire was sent to the evaluator along with the executable file of the software. The evaluator was given a deadline of one week to complete it. If a question or issue arose for the evaluator in any section, he/she would contact the researchers and be given explanations.

In the evaluation process, a comprehensive checklist consisting of 292 questions was used. The questions were organized into separate tabs in an Excel file, with each section having its own sheet. For each question, there were five options available for selection. Three of these options were in the form of checkboxes: "Yes," "No," and "Not Applicable." The evaluator could choose the appropriate option by tapping on it. Selecting "Yes" indicated that the desired criterion existed in the program and had been met, while selecting "No" indicated that the criterion existed but had not been met. Choosing "Not Applicable" meant that the criterion did not exist in the program.

The fourth option in each question was used to assess the severity of any identified problems. If the evaluator answered "Yes" to a question in the previous section, it indicated the presence of a problem in the system. The evaluator was then required to specify the severity of the problem by assigning a number between zero and four. A severity rating of zero indicated disagreement with the existence of a problem, while a rating of one indicated a minor problem that required some time to fix. A rating of two indicated small problems with low priority, a rating of three indicated significant problems with high priority, and a rating of four indicated catastrophic problems that needed to be resolved before the release of the system.

The last option provided space for the evaluator to provide additional comments or descriptions for each question if they had any. This allowed the evaluators to provide further insights or explanations regarding their assessments. By utilizing this comprehensive checklist and its corresponding options, the evaluation process aimed to gather detailed feedback on the system's usability and identify any existing problems or areas for improvement.

## Results

The aim of the current study was to design and develop a serious game-based cognitive rehabilitation system that was implemented in 4 stages. First, the structure and content of the system and games were determined. Then the system was designed and implemented and finally the usability evaluation was done. The findings of each of these steps are shown following.

### Determine the structure and content

This stage was first done by identifying and categorizing existing studies and games for cognitive rehabilitation in the field of attention. At the end of this step, 21 games were extracted from 30 reviewed articles, which were compared from different dimensions. The findings of this step have been previously published in the authors' previous paper [32]. The focus group meetings involved the participation of seven experts from various fields who discussed a range of topics related to the research. The following table shows the demographic information of these participants (Table 1).

**Table 1:** Demographic information of the participants in the focus group meetings

Specialized field of participants	Frequency	Gender		Age average	Experiences average
		Male	Female		
Cognitive rehabilitation	2	0	2	43/5	13/5
Health informatics	2	0	2	38/0	8/5
Game developer	3	3	0	28/3	7/6
**Total**	**7**	**3**	**4**	**36/6**	**9/8**

A part of the results of previous step was presented at the beginning of the focus group meeting as an introduction and familiarization. In this way, everyone in the meeting got to know the purpose of the study and the current state of game development inside and outside the country. According to each topic raised by the facilitator, comments were received by the participants, some of which are shown in the Table 2.

**Table 2:** Some of the comments received from participants on each topic

Num	Topic	Comments
1	In what dimensions do patients with attention deficits need to improve?	"… in order to return to daily life faster and to perform activities that were previously able to do, it is better to consider rehabilitation functions that are in line with returning to daily life. Activities such as navigating the subway or bus, driving, cooking, withdrawing money from the bank teller, making purchases from the supermarket and holding a party."
2	How do we know that games are evidence-based?	"… it is recommended to use existing standard tasks for the assessment and rehabilitation of attention in the design of games. These should be implemented as games so that they are based on evidence and can be accepted as a scientific work."
3	What trainings and methods are appropriate for attention rehabilitation in these patients?	"… Considering that patients with TBI are often between the ages of 20 and 40 and have a low level of literacy, it is recommended that the content of the games be very simple and understandable and can be played by illiterate people as well."
4	What is the level of complexity of the games?	"… due to spatial orientation problems in patients, it is better to use 2D games for them. "
5	What kind of camera angle should be used for the game?	"… Among the camera angles, it seems that the top-down and isometric type is suitable for these patients."
6	What game platform should be selected?	"… Considering the slow speed and high cost of the internet in Iran, creating a game on the web is not recommended at all. For this reason, it is suggested to create a software that can be installed on smartphones and only to send data to the server and data display for the doctor need internet."
7	What should be chosen as the main character in the game?	"… My suggestion is to use a postman character who has a series of missions along the way and each of these missions must be completed. During these missions, points such as coins or an amount of money will be given to the postman."

**Fig. 2:**
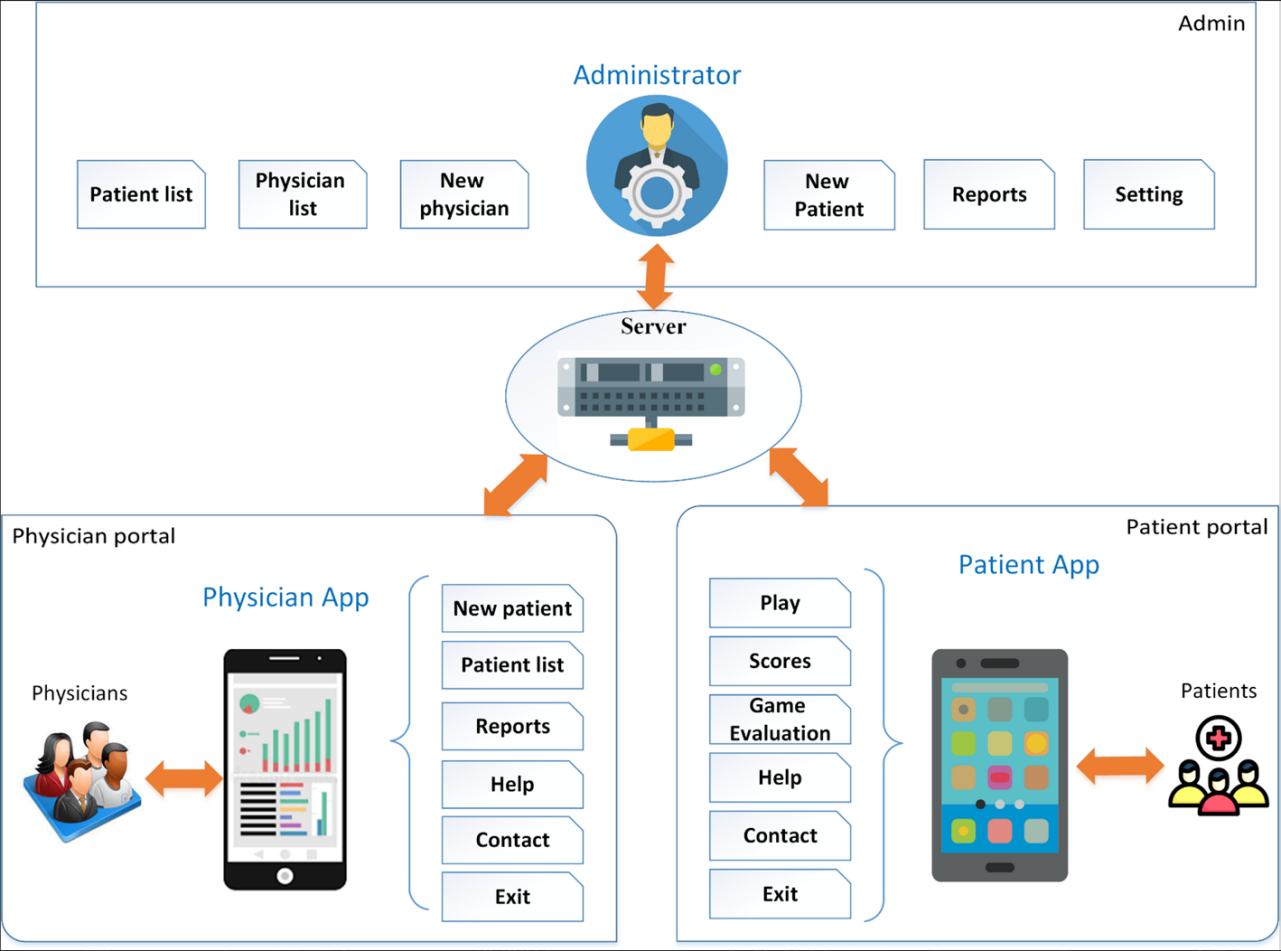
Conceptual model of attention rehabilitation system

Based on the outcomes of the focus group meetings, a conceptual model of the system was designed, as illustrated in Fig. 2. As can be seen, three different types of users were determined for this system. Admin, physician and patient portal. Admin is responsible for creating and managing profiles of physicians and patients. The menus available to this person can be seen in the top box of the admin. The second box is related to the physician's portal, which is responsible for managing patients. Each physician has a separate profile that only has access to his patients. The third box is the patient portal, which is given access to the patient by the physician. The intended platform for all three users is under the mobile application.

In line with the focus group results, it was determined that the postman character would be selected for all the games within the system. The postman character was assigned the task of completing 10 missions or mini-games during gameplay. Each mission was designed based on standard tasks. For example, the first mission is taken from the Trail Making test. This test has two different versions. In the first version, the participant has to draw a line connecting consecutive numbers from 1 to 25. In the second version, the participant connects numbers and letters in a progressive alternating sequence, 1 to A, then A must be connected to 2, 2 to B, and so on. The choice of which tasks to select and what the mission of each task should be was done by experts in the meeting. It was also tried to include all subcategories of attention deficit [41]. The chosen game genre for the system was 2D casual, emphasizing a relaxed and accessible gaming experience. Table 3 provides an overview of all the tasks and their corresponding missions within the system.

**Table 3:** Final missions obtained from meetings with equivalent missions

Num	Tasks	Missions	Types of attention
	Trail Making Test [42]	The postman must move from mailboxes 1 to 10 and pick up the letters	Alternating attention
	Counting a character [14]	Among the packages in the warehouse, count the packages with the first letter of the person's name on it	Sustained attention
	Go/No Go [43] Sustained Attention to Response Task (SART) [44] Continuous performance tests (CPT) [45] Attention Network Test (ANT) [46] Sky search subtest TEA [47]	Among the packages that appear one by one, you must pick up only the packages with a specific color and leave the rest of the packages aside	Sustained attention Selected attention
	Paced Auditory Serial Addition Test (PASAT) [48]	There are loads moving on the freight rail. A number is written on each of the bars. The person must add the number written in the last two packets together and choose the result from among the options	Sustained attention Divided attention
	Walk, Don't Walk subtest TEA [49]	Smoke is everywhere. If the postman hears the sound of movement, it means to move, and if he hears the sound of stop, it means not to move	Sustained attention
	Erikson flanker task Visual Elevator subtest TEA [49] Creature counting subtest TEA [49]	based on the arrow in the middle of the picture, the person chooses the right or left key	Selected attention Alternating attention
	Wisconsin Card Sorting Test (WCST) [50]	One package at a time appears on the rail. Player should choose the package similar to this package in terms of color, geometric shape or number of shapes from among the options	Focused attention
	Stroop test (ST) [51]	The person must choose the correct route according to the color of the delivered package	Selected attention
	Telephone search subtest TEA [49]	During the route, there is a need to call different organizations, such as the emergency or fire department, for which the person must find the number from the phone book	Selected attention
	Opposite world subtest TEA [49]	In some stages of the game, everything is reversed. One has to press the left key to move to the right	Alternating attention

According to the opinion of the experts who participated in the meeting, 16 tasks in different subcategories of attention were selected to make the missions. These includes: Trail Making Test, counting a character, Go/No Go, Sustained Attention to Response Task (SART), Continuous performance tests (CPT), Attention Network Test (ANT), Sky search subtest TEA, Paced Auditory Serial Addition Test (PASAT), Walk, Don't Walk subtest TEA, Erikson flanker task, Visual Elevator subtest TEA, Creature counting subtest TEA, Wisconsin Card Sorting Test (WCST), Stroop test (ST), Telephone search subtest TEA, Opposite world subtest TEA. The tasks were chosen to cover all 5 main subcategories of attention including alternating, sustained, selected, divided and focused attention.

### System design

In this section, various diagrams and documents were created based on the outcomes of the focus group meeting. These include a context diagram, data flow diagram (DFD), use case diagram, scenario tables, class diagram, and a sequence diagram. Furthermore, a one-sheet, ten-pager, and game design document (GDD) were prepared to provide a comprehensive understanding of the system. The game takes place in a 2D environment. Each mission has its own mechanics. But the main mechanics of the game are driving, choosing, picking and calling.

### System development

**Fig. 3:**
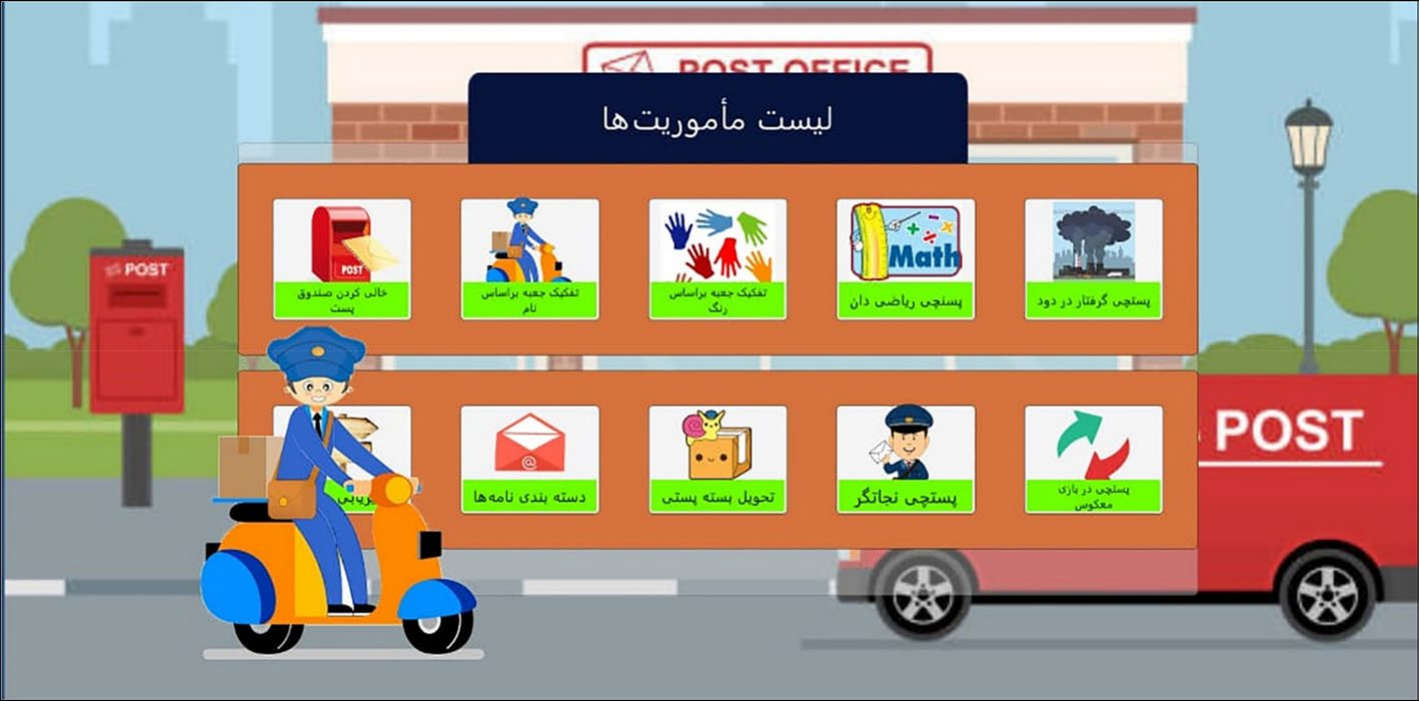
Main page of missions in the patient application

Based on the Game Design Document (GDD), the game environment, characters, and graphic components of the user interface were designed. Following this, missions were created, and the game's user interface was developed for both the therapist and patient versions. The therapist's version of the game's user interface includes sections for recording and editing patient information, a dashboard for viewing patient scores, and sections related to results. Additionally, the main page for selecting games, sound and music related to each mission, and the entirety of the game were added. Figure 3 represents the main page of the patient version, showcasing the various missions available for selection.

As mentioned, the game story is about a postman who has to complete various missions during the game. During the game, the postman gains more capabilities and can perform more advanced missions. At each stage, he is given a star reward for success. In some missions, the postman rides a post office car. He has to move and transfer letters and postal packages during the game. In this way, sometimes it is necessary to increase the ability and skill in driving and navigation. Daily, various missions are assigned to him, such as delivering letters, choosing the package in the warehouse, moving packages, correct routing, etc. In Table 4, the images of all the games are presented along with their descriptions.

**Table 4:** The image of all the games with their descriptions

N	Images of mini games	Descriptions
1		**First mission (Emptying the mailbox):** The postman must move from mailboxes 1 to 10 and pick up the letters
2		**Second mission (Separation of boxes by name):** Among the packages in the warehouse, count the packages with the first letter of the person's name on it
3		**Third mission (Separation of boxes by color):** Among the packages that appear one by one, you must pick up only the packages with a specific color and leave the rest of the packages aside
4		**Fourth mission (Mathematician postman):** There are loads moving on the freight rail. A number is written on each of the bars. The person must add the number written in the last two packets together and choose the result from among the options
5		**Fifth mission (Postman trapped in smoke):** Smoke is everywhere. If the postman hears the sound of movement, it means to move, and if he hears the sound of stop, it means not to move
6		**Sixth mission (Navigation in the city):** Based on the arrow in the middle of the picture, the person chooses the right or left key
7		**Seventh mission (Classification of packages):** One package at a time appears on the rail. Player should choose the package similar to this package in terms of color, geometric shape or number of shapes from among the options
8		**Eighth mission (Postal package delivery):** The person must choose the correct route according to the color of the delivered package
9		**Ninth mission (Rescue postman):** During the route, there is a need to call different organizations, such as the emergency or fire department, for which the person must find the number from the phone book
10		**10th mission (The postman in the reverse world):** In some stages of the game, everything is reversed. One has to press the left key to move to the right

In some missions, the touch arrows designed on the mobile screen give the player the ability to control the main character. For example, in the first mission, the patient must guide the postman's car with the help of arrows on the mobile screen and empty the mailboxes from number 1 to 10 in order. For each mission, a series of data is saved to evaluate and display the player's performance by the physician. The data collected in this mission includes the number and percentage of errors per game, the number and percentage of success per game, points per game, the total number of attempts and the duration of the game.

### Evaluation

The research sample for evaluating the usability of the attention rehabilitation system consisted of 5 experts who possessed proficiency in usability issues. The evaluation utilized a checklist that assessed the system's features based on 13 different principles, comprising a total of 292 questions. Upon collecting the completed checklists, the data were analyzed, revealing a set of problems associated with each component of the system. These identified problems will be addressed and corrected in the subsequent version of the system. The findings indicated that the highest percentage of problems to the total number of items in a domain was related to privacy (number = 3 from 3; 100 percent), help and documentation (number = 13 from 23; 56.52 percent), and aesthetic and minimalist design (number = 6 from 12; 50 percent). Furthermore, the most severe problems were identified in the areas of privacy (mean = 3.23), error prevention (mean = 2.68), flexibility, and minimalist design (mean = 2.63). The overall evaluation results are presented in Table 5*.*

**Table 5:** Result of heuristic evaluation

Number	Title of principal	Total questions	Number of problems reported	Percentage of problems reported	Average severity of problems
1	Visibility of system status	29	9	31.03	1.18
2	Match between the system and the real world	24	4	16.67	1.60
3	User control and freedom	23	9	39.13	2.00
4	Consistency and standards	51	24	47.06	1.91
5	Help users Recognize, diagnose, and recover from errors	21	9	42.86	2.63
6	Error prevention	15	5	33.33	2.68
7	Recognition rather than recall	40	17	42.50	2.04
8	Flexibility and minimalist design	16	7	43.75	2.63
9	Aesthetic and minimalist design	12	6	50.00	1.33
10	Help and documentation	23	13	56.52	2.28
11	Skills	21	8	38.10	1.88
12	Pleasurable and respectful interaction with the user	14	2	14.29	1.50
13	Privacy	3	3	100.00	3.23
**Total**		**292**	**116**	**39.73%**	**2.07**

## Discussion

Based on the findings, the system model consists of 10 games, which have been designed based on 16 standard tasks or tests. This is because the research team had to merge some tasks in the first phase due to cost and time constraints and cover one mission for several tests. The choice of the casual genre for the games was driven by the need to align them with the standard tasks. The postman character was selected as the protagonist of the game. Throughout the gameplay, the postman character is required to complete various tasks and receive rewards for their successful completion. Bonuses are stars that are awarded to a person during the game. The more stars, the higher the score.

According to the reviewed studies, it seems that few comprehensive softwires for cognitive rehabilitation in patients with brain injuries have been created that both patients and physicians can access everywhere [52]. CogMed and Brain HQ are two famous softwires that were created specifically for cognitive rehabilitation in patients with brain injuries. BrainHQ is an online cognitive training system where each user can be monitored during the entire training [52]. Unlike most of the existing games [53–56], the current system had the ability to be installed on smart phones in two versions for physician and patient, and therefore it is available everywhere and with any quality of internet access.

For this study, the focus group method was employed, as it allowed for a relatively quick assessment of the experiences and priorities of different groups of professionals. Focus groups are valuable in gaining insights into the target population affected by the intervention, understanding their thought processes, and learning about their behaviors. They serve as a means to identify requirements, gather ideas, and gather thoughts from the target groups. Overall, the focus group method was an effective approach in this study to gain a comprehensive understanding of the target population's needs and perspectives [57]. In Mercado et al.'s [58] study, the user-centered research process was employed, similar to the current study. They utilized a combination of qualitative research methods, including interviews and observation, along with the think-aloud method, to develop their system [58].

Martin et al. [59] also used a qualitative study with a user-centered approach to gathering requirements. They organized two focus group meetings to gather needs, including therapists and experts. Of course, sometimes the large number of people in the meetings makes it difficult to understand and make a single decision. For this reason, the number of people present in the meeting is recommended to be between 6 and 12 people [60]. Various studies emphasize the existence of people with different expertise in developing technological models [59, 61, 62].

Our study selected the use of games related to people's daily lives. In Giglioli's [63] and Gamito et al., [31] studies, similar to the current study, the trains assigned to the person were related to daily life activities [31, 63]. The games created by the study of Baranyi et al., [29] and Vourvopoulos et al., [30] were in line with the daily life of the patients. This issue can be useful for returning to activities in patients with brain injuries. Of course, as mentioned in the focus group meetings, attention should also be paid to the simplicity and comprehensibility of the games. The character of this game was chosen as a postman. By utilizing it, the storyline of various mini-games can be observed, and patients with brain injuries do not have a bad background and mentality toward this character. During the meetings, the character of a taxi driver, a policeman, or the use of an animal character such as a frog, a hedgehog, a rabbit, or a turtle was also discussed. However, each of them withdrew from the discussion due to reasons such as the bad mentality of the patients toward this character, recalling the accident's memory, and the character's childish nature. In Baranyi et al., [29] study, various videogames such as making a cup of coffee, cooking, and taking a bath were designed in relation to people's daily lives. The name of this game was My Daily Routine [29]. Another game was also developed by Vourvopoulos et al., [30] called RehabCity. This game was a kind of city simulation that includes streets, moving cars, sidewalks, parks, buildings, etc. They chose four common places of interest (pharmacy, bank, post office and supermarket) that encourage users to interact in a safe environment [30].

As mentioned in the explanations of the focus group meetings, the simplicity of the games must also be taken into consideration. In Giordani et al.'s [64] study, it is emphasized to observe the principle of simplicity of mechanics in design. Because the persons who use this application have little experience in using modern technologies. A very simple click of a mouse or a touch screen of a smartphone that provides immediate and uncomplicated feedback in the initial stages of work, and as the games continue, the complexity slowly increases, is one of the solutions for the simplicity and comprehensibility of the game. More complex game concepts, such as player avatars, or drag-and-drop actions are uncomfortable for many people with cognitive difficulties [64].

The unity game engine was also used as the main tool for building the system. The required graphic elements and components were also created using Adobe Photoshop 2021. The software was created on a mobile-based platform to be easily accessible for both the patient and the therapist. According to research, the use of smartphones is high among young people, and smartphone owners use an average of 10 apps per day and 30 apps per month [65]. It has been used as an educational intervention for cognitive rehabilitation in populations with attention deficit disorder [66] and elderly people [67]. Both of these studies used the same brain-computer interface (BCI) intervention to enhance users' attention skills through a 3D video game as part of an attention training program [66, 67]. Giordani et al., [64] in a similar current study, developed a game with a tablet or smartphone platform and stated that these platforms work better both in motivating children and simplifying the response mechanisms. In the study of Baranyi et al., [29] like the current study, the games were placed in a portal that has other modules [29].

To evaluate the usability of the system, Pierotti & Nielsen's [39] checklist was used. The research findings showed that the highest percentage of problems were related to privacy, help and documentation, and aesthetic and minimalist design. The most serious problems were in the sections related to privacy, error prevention, flexibility, and minimalist design. With the increasing complexity of new technologies, the usability evaluation of these technologies, in the context of human–computer interaction and user interface design, becomes more important. Even the most innovative products risk failing completely if end users cannot fully engage with the technology due to user interface issues. As a result, product designers increasingly focus on usability testing at the prototype stage to identify design issues and prevent successful user interaction with the final product. The field of serious games is a good example where usability issues should be given special attention [68]. As stated by McLeod and Ranger [69], various methods are commonly used to evaluate the usability of systems [69]. One technique that has the potential to be useful in evaluating game prototypes is heuristic evaluation. It is a suitable usability method widely accepted in software design and has advantages such as flexibility and low cost [70].

Heuristic evaluations are appropriate at any stage of the software development cycle, even after that, and do not require a fully functional prototype [71]. Jacob Nielsen proposed the usability heuristic method for general software design. This method is a useful tool for designing most software [70]. Unlike the current study, Fernandez et al. [72] used the SAS questionnaire to evaluate the applicability of video games [72]. It seems that more methods are needed to evaluate the usability of games. Especially in the Persian language, there was no questionnaire specifically localized to the field of serious games. The current research only evaluated usability. Nevertheless, in the next steps, studies will be conducted on patients, families, and therapists to check the final performance of the system.

## Conclusions

In the current study, several considerations were taken into account to address the limitations and meet the needs of the patients. To ensure ease of understanding, the games were created in a casual 2D genre and designed as mini-games. These mini-games were based on daily life activities and incorporated a story element. The system caters to three types of users: the therapist, the patient, and the system administrator. The development of the games was guided by standard tasks of the attention domain, resulting in the inclusion of 16 standard tasks as game models. The character of the game was chosen as a postman, and the prototype of the system featured ten missions for the player to complete. Considering the specific needs of patients with traumatic brain injury (TBI), the game was designed to be simple and understandable, taking into account their age and cultural background. In terms of evaluating the system's usability, the Nielsen heuristic method by Pierotti & Nielsen's checklist was employed. This method is a cost-effective and valid tool for assessing usability, saving time and resources. The evaluation results highlighted areas that require improvement, including privacy, help and documentation, aesthetic and minimalist design, and error prevention. These areas will be given more attention in the next version of the system, with the aim of strengthening these aspects. Following the refinement process and addressing the identified issues, the system will undergo evaluation by patients in a real environment, allowing for further feedback and assessment. This step is underway, the results of which will be published soon. The current developed system is a prototype, and in the future, more modules and facilities will be added to the system, such as automatic determination of missions according to the person's ability, personalization the user interface, and the use of intelligent algorithms to identify the behavioral patterns of patients.

## Data Availability

All data and material are available under the requirement to the corresponding authors.

## Abbreviations

TBI: Traumatic Brain Injury
GDD: Game Development Documents
BPG: Brain Powered Games
DFD: Data Flow Diagram
UML: Unified Modeling Language
